# Rediscovering Psilocybin as an Antidepressive Treatment Strategy

**DOI:** 10.3390/ph14100985

**Published:** 2021-09-28

**Authors:** Rene Zeiss, Maximilian Gahr, Heiko Graf

**Affiliations:** Department of Psychiatry and Psychotherapy III, University of Ulm, Leimgrubenweg 12–14, 89075 Ulm, Germany; Maximilian.gahr@uni-ulm.de (M.G.); heiko.graf@uni-ulm.de (H.G.)

**Keywords:** psilocybin, psilocin, review, depression, antidepressant therapy

## Abstract

There has recently been a renewal of interest in psychedelic research on the use of psilocybin in psychiatric treatment and, in particular, for the treatment of major depressive disorder (MDD). Several state-of-the-art studies have provided new insight into the mechanisms of action of psilocybin and its therapeutic potential. Nevertheless, many questions remain unanswered. With this review, we provide an overview of the current state of research on the potential mechanisms of psilocybin, its antidepressant potential, and the associated risks and adverse effects, to provide an update on a controversial topic discussed in psychopharmacology. A database search was conducted in Medline including articles on psilocybin over the period of the last 20 years. Despite the promising progress in understanding the mechanisms of psilocybin, the exact antidepressive mechanism and the role of the psychedelic experience remain elusive. The studies included in this review found high treatment effect sizes for psilocybin as an antidepressant. However, the results must be regarded as preliminary due to several limitations. Although the current studies observed no severe adverse events, several questions regarding safety and utility remain and must be subject of future research.

## 1. Introduction

Major depressive disorder (MDD) is one of the most commonly diagnosed mental disorders and one of the largest contributors to the global disease burden [1]. Numerous clinical and neuroscientific efforts have been undertaken in recent decades to provide a better understanding of the etiopathology of MDD, yielding important insights into abnormalities in genes, neurochemical and neuroendocrine systems, functional and structural brain anatomy, inflammatory processes, and cognition [2,3]. The most influential neurobiological discoveries have probably been neurotransmitter-related abnormalities within the monoaminergic/catecholaminergic system, including the neuromodulators serotonin, noradrenalin, and dopamine. Accordingly, one of the main strategies in the pharmacological treatment of MDD comprises the reuptake-inhibition of monoamines, in particular serotonin and/or noradrenaline. Other antidepressants directly target monoamine (e.g., serotonin receptors by vortioxetine) or other receptors (e.g., melatonin 1 and 2 receptors by agomelatine) [4].

However, despite the efficacy of several pharmacological and non-pharmacological treatment options for MDD, there is still a high rate of treatment-resistant depression [5,6], supporting the need for further and new treatment options for MDD [7,8,9,10]. After decades of relative obscurity, scientific research is recently focusing on the use of psychedelics as a potential approach for the treatment of psychiatric disorders [11,12,13,14]. In addition to MDMA-assisted therapy as a treatment option for PTSD [15], there is preliminary evidence for the antidepressant efficacy of psilocybin, presumably due to its serotonergic effects.

Psilocybin is a tryptamine derivate that occurs naturally in numerous mushrooms such as *psilocybe mexicana* and *psilocybe cubensis*. Albert Hofmann isolated psilocybin and its main metabolite psilocin from these two species for the first time in the 1950s [16]. Psilocybin-containing mushrooms have been used in religious rituals for centuries, if not millenia [17,18]. Some of the earliest written records stem from the Spanish friar Bernardino de Sahagún, who noted the sacred mushrooms used in Aztec spiritual acts. They were called teonanacatl or “God’s Flesh” [19]. Psilocybin was first introduced into the modern Western world by Robert Gordon Wasson after attending a Mazatec ritual called “Velada”. In 1957, he shared his experiences in a “Life” article called “Seeking the Magic Mushroom” [20]. During the 1950s and 1960s, there was also an intense period of research on the use of “classical” psychedelics resulting in more than 1000 scientific papers, including data from over 40,000 participants [21]. LSD in particular, among other psychedelics, was used in the treatment of depression, anxiety, or alcohol disorders [21].

However, this initial wave of research on psychedelics indicating antidepressant effects was followed by a decline in interest. The increasing popularity and recreational use of psychedelics, inter alia among followers of protest movements, the political atmosphere, and incidences such as the “Harvard drug scandal”, cumulated in the illegalization of psilocybin and other psychedelics in the United States in 1968 [22]. Psilocybin was and is still categorized as a Schedule I drug in the “Convention on Psychotropic Substances”, a United Nations treaty designed to establish a control system for psychotropic substances. Accordingly, research became considerably complicated and psilocybin was supposed to create “a serious risk to public health” and was not considered to have a “known therapeutic benefit” [23]. The legal status of psilocybin remains in flux depending on the country or state. Although the possession and sale of psilocybin remains illegal in the US according to federal law, several states (e.g., Colorado and California) partially decriminalized the substance. In Oregon, a ballot was in favor of legalizing psilocybin, including plans to allow its therapeutic use [24]. The legal status of psilocybin also varies across the EU. Although some countries list hallucinogenic mushrooms as a controlled substance, making its cultivation, sale, and possession illegal (e.g., in Germany), others consider cultivation as legal as long as it is not for drug abuse (e.g., Austria) [25].

After decades of hibernation, psilocybin and other psychedelics have recently received increasing scientific attention as potential therapeutic approaches for various mental disorders, e.g., tobacco addiction [26], alcohol dependence [27], anxiety in cancer patients [28,29], obsessive compulsive disorder [30], and depression [31]. In particular, the pivotal role of psilocybin as an antidepressant treatment option has captured media’s attention after the FDA granted a “Breakthrough Therapy Designation” to a psilocybin trial on MDD [32].

These studies have not only attracted scientific and clinical attention, but also the interest of the public, and this topic is increasingly represented in public media. Thus, clinicians will be increasingly engaged with this topic. In this review, we provide an overview of the current literature to provide a better understanding of the non-pharmacological and pharmacological mechanisms of action and caveats associated with the use of psilocybin.

## 2. Materials and Methods

We conducted a database search in Medline, from 2000 until present (06/2021). The search was performed in June 2021 and abstracts were screened independently for relevance by RZ and HG. We used the following terms as the search strategy: “(“Psilocybin”[MeSH]) AND ((((((“Depression”[MeSH Terms]) OR (“Depressive Disorder”[MeSH Terms])) OR (“depressive disorder, treatment resistant”[MeSH Terms])) OR (“Dysthymic Disorder”[MeSH Terms])) OR (“depressive disorder, major”[MeSH Terms])))”, “((“Psilocybin”[Mesh]) AND (“adverse effects”[Mesh])” and (“Psilocybin”[Mesh]) AND “Neuroimaging”[Mesh]).

## 3. Discussion

### 3.1. Mechanism of Action

#### 3.1.1. Pharmacological, Neurobiological and Neuroimaging Findings

Psilocybin and its main metabolite psilocin act through a variety of mechanisms which are not yet fully understood [33]. Despite its affinity to several serotonin receptors, such as the 5HT_2B_, 5-HT_2C_, 5HT_5A_, 5-HT_1A_, 5-HT_1D_, 5-HT_1E_, 5-HT_6_, and 5-HT_7_-receptors [34,35,36,37,38], the most important antidepressant mechanism of action is thought to be the (partial) agonism at the 5-HT_2A_ receptor [11,39], relevant in the pathway implicated in suicidality and depression [40,41,42]. The pivotal role of the 5-HT_2A_ receptor is further supported by the observation that the psychotomimetic effects of psilocybin can be impeded by the administration of 5-HT_2A_ antagonists, e.g., ketanserin [43]. Further evidence for the pivotal role of the 5-HT_2A_-receptor for the antidepressive effect stem from a double-blind study with 17 healthy participants. The processing of negative emotional stimuli and mood states was altered by the administration of psilocybin (215 μg/kg) and a previously found diminished recognition of negative facial expression could be blocked by the administration of 50 mg ketanserin [44]. Moreover, in a PET study on eight healthy participants taking doses between 3 and 30 mg of psilocybin, the 5-HT_2A_ occupancies were found to be dose related [45]. The 5-HT_2A_ occupancies were up to 72% and correlated with the subjective intensity and psychedelic effect [45].

One proposed mechanism for the antidepressant effect of 5-HT_2A_ agonists is the downregulation of the 5-HT_2A_-receptors [46]. However, this downregulation of 5-HT_2A_ density by psilocybin does not appear to be a lasting effect. In a recent PET study on healthy volunteers, the 5-HT_2A_ binding of psilocybin one week after administration did not differ significantly from that of the baseline [47]. Similar effects were observed in an animal model. A single dose of psilocybin (0.08 mg/kg) led to a reduced 5-HT_2A_ density in the hippocampus and the prefrontal cortex after one day that was not observed after seven days post-injection [48]. Nevertheless, the same study demonstrated a higher hippocampal synaptic vesicle protein 2A density that remained significantly higher 7 days post-treatment, suggesting an increased persistent synaptogenesis [48].

In addition to modulating monoaminergic levels in the synaptic cleft, there is evidence for considerable effects of psilocybin on neuroplasticity and neurogenesis, relevant in the pathophysiology of mood disorders [49,50]. In a mouse model, several psychedelics including psilocybin promoted an increase in dendritic arbor complexity and dendritic spine growth, and stimulated synapse formation [51]. However, long-term data considering structural alterations in humans by psilocybin are scarce. Recent evidence from animal models suggest that the antidepressant effects of psilocybin apparently cannot be fully explained by 5-HT_2A_ agonism. In a study on the effects of psilocybin on chronically stressed mice, an increase in hedonic responses was found under psilocybin in addition to strengthening of excitatory hippocampal synapses, although the 5-HT_2A_ antagonist ketanserin was administered a priori [52].

In addition to these pharmacological and animal studies, various neuroimaging studies have investigated the effects of psilocybin on neural network activations to elucidate its antidepressant mechanisms, indicating considerable differences between acute and short-term post-treatment effects. Most of these studies focused on intrinsic neural network architecture and, in particular, on the Default Mode Network (DMN) considering an increased and ineffective suppression of functional connectivity in this network in MDD [53]. Carhart-Harris et al. [54] investigated the acute effect of psilocybin in 15 healthy participants and demonstrated a significant reduction in functional network connectivity within the DMN after intravenous administration of psilocybin compared to placebo. An open-label study by the same research group in depressed patients receiving psilocybin, observed a decrease in cerebral blood flow (CBF) within the amygdala, and an increase in DMN integrity, one day after administration of 10 and 25 mg, respectively, of psilocybin [55]. A further analysis also considered the fMRI data of a phase 2 trial on psilocybin vs. escitalopram, focusing on neural network modularity. Although this preprinted study is currently still under review and results have to be considered as preliminary, the authors observed a significantly reduced brain network modularity [56]. According to these neuroimaging findings with differential acute and short-term post-treatment effects, one hypothesis regarding the mechanism of psilocybin is that the 5-HT_2A_-agonism may provoke an “entropic brain state” suggestive for “resetting” brain networks, particularly within DMN [14].

However, the exact underlying mechanisms remain unclear. One study investigated the acute effects of psilocybin in 15 healthy subjects and assumed a pivotal role of alterations in 5-HT_2A_ receptor signaling in the claustrum with consecutive decreases in DMN activity [57]. Another task-based fMRI suggests that the antidepressive efficacy of psilocybin is due to an increase in neurofunctional responsiveness within the amygdala to emotional stimuli, which allows patients with MDD to work through them [58]. The increase in neural activity within the amygdala correlated with treatment efficacy. This initially appears counter intuitive because a decrease in amygdala hyperactivity has usually been associated with SSRI treatment in depression [59,60]. A further analysis of these data also showed a decrease in functional connectivity between the right amygdala and the ventromedial prefrontal cortex [61].

Whereas most of these studies investigated acute effects of psilocybin on functional network connectivity, one study on 10 healthy volunteers evaluated the effects of a single psilocybin administration on functional connectivity of the executive control network (ECN) one week and three months after intake. The significant decrease in ECN functional connectivity after one week of administration of psilocybin was not evident after three months [62].

In conclusion, despite the considerable efforts to elucidate the molecular and neurofunctional mechanism behind the antidepressant effects of psilocybin, they are still not fully understood. Furthermore, there is increasing evidence that the antidepressant efficacy of psilocybin is not only due to molecular or neurofunctional mechanisms, but also strongly depends on other non-pharmacological parameters.

#### 3.1.2. Set and Setting: Nondrug Parameters of Psychopharmacology and the Role of the Psychedelic Experience

The importance of “set” and “setting” in psychedelic-assisted therapy was emphasized by Timothy Leary in the 1960s [63,64]. Whereas “set” refers to more internal factors, such as the personality, expectations, and state of mind of an individual when entering a psychedelic session, “setting” describes external factors, such as the physical and social environment of the session [64]. A thorough review of the topic of set and setting, in addition to its history, can be found at Hartogsohn 2016 and 2017 [64,65]. Of note, “set”, “setting” and the type of psychedelic experience apparently may not only have an impact on the acute experience, but also on the long-term outcome of psilocybin-assisted therapy. In a trial involving 20 patients with treatment-resistant depression who received two doses of psilocybin in two separate sessions, the quality of the acute experience during the session appeared to be of major importance for long-term changes [66]. A high “mystical-type experience” was predictive, whereas anxiety and impaired cognition correlated negatively with a positive long-term outcome [66]. A study evaluated potential predictive variables for the response to psilocybin in 261 healthy volunteers who underwent a total of 409 psilocybin administrations. They found that a high score in the personality trait of absorption, being in an emotionally excitable and active state immediately before drug intake, and the experience of few psychological problems in preceding weeks, were strongly associated with higher “mystical-type experiences” and pleasant experiences [67]. In contrast, young age, emotional excitability, and an experimental setting (e.g., neuroimaging) predicted unpleasant or anxious reactions to psilocybin experiences [67]. A prospective study using a web-based data collection to predict responses to psychedelics not limited to psilocybin, also found a positive effect on well-being that was associated with a “mystical-type experience”. In addition, having clear intentions and the feeling of being ready for the experience had a positive impact on “mystical-type experience”, supporting the importance of “set” and “setting” [68].

Despite the association between the “mystical-type” experiences and the therapeutic benefit, it remains unclear whether this link is merely correlational or causal. Some authors argue that the psychedelic effect may represent a marker for the responsiveness of the 5-HT_2A_ receptor, and thus for neuroplasticity and psychoplastogenic effects [69].

A research group developed a biosensor based on the structure of the 5-HT_2A_ to predict the hallucinogenic potential of 5-HT_2A_ ligands. They identified a non-hallucinogenic 5-HT_2A_ ligand and observed antidepressant effects in mice. This antidepressant effect measured by the forced swim test was still detectable one week after a single dose administration, suggesting a long-lasting effect [70].

In line with these observations, another study investigated the effects of tabernanthalog, a non-hallucinogenic 5-HT_2A_-agonist and non-toxic analogue of ibogaine in rodents. In contrast to ibogaine, they observed antidepressant effects of tabernanthalog, a reduction in heroin- and alcohol-seeking behavior, and an increased neural plasticity [71].

**Figure 1:**
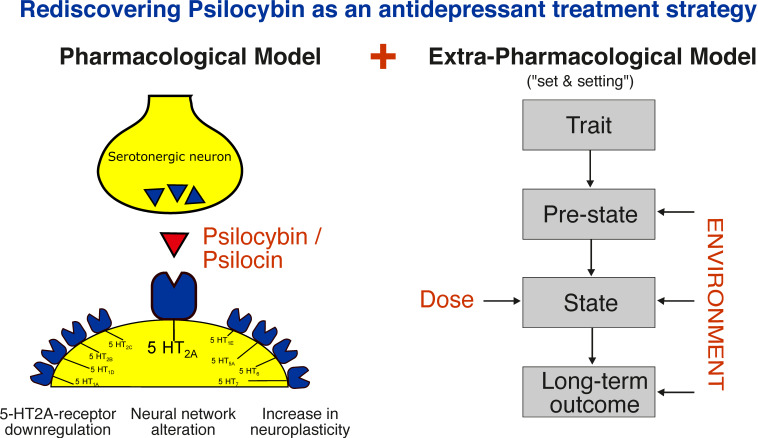
Mechanisms of action of psilocybin: A pharmacological and extra-pharmacological model. Extra-pharmacological model reprinted from ref. [37].

In summary, the importance of the psychedelic experience for the therapeutic effects of psilocybin remains controversial [69,72] and will be a subject for further studies. A graphical summary of the pharmacological and extra-pharmacological model is shown in Figure 1.

### 3.2. Psilocybin in Antidepressive Therapy

Although intense research on the use of psychedelics in the treatment of psychiatric disorders was conducted for a period of about two decades during the 1950s and 1960s, the number of clinical trials addressing psilocybin in the therapy of mood disorders is relatively low compared to the number of trials on LSD [73]. This changed during the current wave of psychedelic research, with an increasing number of studies being conducted, particularly of antidepressive efficacy [74]. Although the detailed mechanism of action of psilocybin still has to be examined in further research, various studies and systematic reviews demonstrate an antidepressant efficacy of psilocybin, as summarized in the following section.

#### 3.2.1. Antidepressive Effects

One of the first studies of the new wave of psychedelic treatments and their antidepressant effect was conducted within a double-blind, placebo-controlled pilot study design on 12 patients with cancer and depressive symptoms. One group was treated with 0.2 mg/kg of psilocybin, and the placebo control group received niacin as an active placebo. In the psilocybin group, BDI-scores [75] improved by 30% one month after treatment. Of note, this improvement in depressive symptoms was still observed in a 6 month follow-up examination [28]. However, these results are limited, not only due to the small sample size; one should also consider that participants fulfilled the diagnosis of acute stress disorder, generalized anxiety disorder, anxiety disorder related to cancer, or adjustment disorder with anxiety related to cancer, but not MDD. In addition, the study design did not include a control group. Nevertheless, similar results on cancer-related depressive symptoms were observed in further studies. Within a crossover design, 29 cancer patients received either psilocybin (0.3 mg/kg) or niacin as an active placebo control. The psilocybin group showed significant improvements in depression scores compared to niacin [29]. In a long-term follow-up of the study after 4.5 years, the surviving participants still showed clinically significant less depressive symptoms than the control group, although the beneficial effects of other therapies during the follow-up period could not be excluded [76]. Another study investigated dose-dependent effects of psilocybin on depressed mood and anxiety in patients with a life-threatening cancer diagnosis within a cross-over design. Depressed mood and anxiety were assessed by the GRID-Hamilton Depression Rating Scale (GRID-HAMD) and the Hamilton Anxiety Rating Scale (HAM-A). Response was defined as at least 50% relative to baseline, and symptom remission was defined as a decrease of at least 50% relative to baseline and a score of 7 or less in both scores. A share of 92% of the patients in the high dose group (22 or 33 mg/70 kg) showed a response after 5 weeks compared with 32% in the low dose group (1 or 3 mg/70 kg). After 6 months, when both groups had received the high dose, the clinical response rate was 78% for depressive symptoms with a remission rate of 65% [77].

An open-label study investigated the effects of two doses (7 days apart) of psilocybin in a sample of 12 participants with treatment-resistant MDD. Treatment-resistant depression was defined as no improvement despite two courses of treatment with antidepressants of different pharmacological classes lasting at least 6 weeks. The study observed a response rate of 67% with full remission (defined as a BDI score of 9 or less) after one week in eight patients, of which five remained in remission at the 3 month follow-up. Within one week after administration, the average MADRS score improved from 31 to 9.7, indicating an improvement from moderate/severe to mild depression, although the study also included psychological support prior, during, and after the psilocybin sessions [78]. The study was later extended to 20 participants and the follow-up period was lengthened from 3 to 6 months. The authors observed significant effect sizes (Cohen’s d) in patients with moderate to severe treatment-resistant depression after short-term assessment (1–5 weeks) of 2.1–2.3 (*p* < 0.001) and after long-term assessment at 3 (d = 1.5, *p* < 0.001) and 6 months (d = 1.6, *p* = 0.004), as measured by the Quick Inventory of Depressive Symptoms (QIDS-SR16). All patients received a moderate (10 mg) and a high dose (25 mg) of psilocybin, but there was no control group [79].

Further evidence on the antidepressant efficacy of psilocybin stems from a randomized waiting list-controlled trial in a total of 27 patients with MDD that received either immediately (*n* = 15) or with a delay of several weeks (*n* = 12) two doses (20 mg/70 kg at the first session; 30 mg/70 kg at the second session) of psilocybin. The severity of depression was assessed with the GRID-HAMD, and only patients with a score of ≥17 were included. The authors observed a significant improvement in depressive symptoms in the immediate compared to the delayed group. After one and four weeks of intervention, respectively, GRID-HAMD-scores were 8.0 and 8.5 (immediate group) compared to 23.8 and 23.5 (delayed group) [80], with significant effect sizes in week 5 and 8 (Cohen’s d = 2.5, *p* < 0.001 and d = 2.6, *p* < 0.001). In total, 71% of the patients underwent a clinically significant reduction in depressive symptoms defined as a GRID-HAMD-score reduction of ≥50% at week one and four after intervention, and 54% of the participants were even in remission (defined as a score of ≤7) after four weeks [80].

It should be considered that each new approach is usually measured against established therapeutical options and a patient-relevant therapeutic additional benefit is of crucial importance. In a recent phase 2 trial, antidepressant effects of psilocybin were compared with escitalopram, a well-established SSRI antidepressant, in a sample of 59 patients. Over a six week period of treatment, no significant differences between psilocybin and escitalopram were observed regarding their antidepressant effects [81]. The adverse events were also similar, and no serious adverse events were reported. Thus, further studies are needed to compare psilocybin and established antidepressant agents, particularly regarding the long-term outcome in severe and treatment-resistant depression and potential adverse effects.

#### 3.2.2. Systematic Reviews and Meta-Analysis

We also included the results of five systematic reviews and meta-analysis. Considering the new interest in psychedelic research and the evolving but nonetheless young field of research on this topic, the number of studies included in these reviews is low, and overlap exists among several of the systematic reviews. In addition, it is notable that some of the studies included in the systematic reviews were introduced in the previous section.

One meta-analysis particularly investigated the effects of psilocybin on anxiety and depression, and included one uncontrolled and three randomized, placebo-controlled studies with a total of 117 participants. The beneficial pre-post effects on anxiety and depressive symptoms in the placebo-controlled studies (*n* = 97 patients) were described as statistically significant with a Hedges’ g of 0.82 to 0.83 [31]. A systematic review on long-term effects of psychedelics in general included nine studies investigating psilocybin treatment in depressed patients. In the majority of patients, a short-term reduction in depressive symptoms was found, which was sustained for the follow-up period of six months, as investigated by five of the included studies [82]. Another systematic review examined the use of psychedelics for the treatment of depression and anxiety. This review included four studies assessing the use of psilocybin for depression [83] and described a significant antidepressant effect of psilocybin that was well tolerated [83].

A meta-analysis on psychedelics for treatment of mood and depressive symptoms investigated 12 studies, of which eight studies considered psilocybin [84]. They found a significant, moderate clinical effect size for psilocybin regarding the reduction in depressive symptoms in patients suffering from a mood disorder.

### 3.3. Adverse Drug Reactions and Harm Potential

#### 3.3.1. Physical and Mental Harm Potential

Although psychedelics are still categorized as a “Schedule 1” drug with “serious risk to public health”, data on physical harm resulting from this group of drugs are scarce. Psilocybin’s lethal dose (LD) 50 value is 280 mg/kg in rats, which is roughly 17 kg of fresh mushrooms in adult humans [85]. As far as it is known by current research, psilocybin has no or very low potential for physical dependence or drug withdrawal [11,86].

Nevertheless, controversy exists regarding psychological side effects and the negative impact of psilocybin on mental health. An often-mentioned but rare major safety concern is the so-called “Hallucinogen Persisting Perception Disorder” (HPPD). Here, patients experience hallucinogen-induced effects, such as visual anomalies or visual hallucinations after the actual drug effect has worn off. The prevalence of HPPD cannot be quantified in detail but is considered low, and most of these cases are reported for LSD and more often with recreational use. However, there are also case reports of HPPD after the intake of psilocybin [11,87].

In a risk assessment on psilocybin performed by CAM, an advisory board providing science-based advise about recreational drugs, the substance was considered to be “relatively safe”, with few adverse effects described, none of which were severe [85]. However, potential risks of psychedelics, and particularly psilocybin, in recreational use cannot be equated with potential adverse drug reactions in their medical use.

Population studies focusing on lifetime recreational psychedelic use and associated health problems have mentioned that recreational use of psychedelics may reduce psychological distress and suicidality [88,89]. These findings must be regarded with caution, because neither study was able to entirely control for other variables, such as the use of other substances. For example, lifetime use of other illicit substances such as marihuana was associated with past-year suicidal thinking and past-month psychological distress, and 97.7% and 97.8%, respectively, of the psychedelic users also reported lifetime marihuana use [88,89]. Furthermore, several case reports describe harmful behavior in particular when psychedelics were consumed together with other psychotropic substances, e.g., alcohol [90,91,92]. An online survey about the single most challenging experience with recreational psilocybin use revealed that 11% put themselves or others at risk of harm, 7.6% sought treatment because of enduring psychological problems, and three cases were associated with the onset of enduring psychotic symptoms [93]. However, one may assume a higher number of unreported cases.

#### 3.3.2. Adverse Drug Reactions in Therapeutic Settings

The studies included in this review investigating the use of psilocybin as an antidepressive agent reported no serious adverse events. None of the systematic reviews mentioned described any serious adverse events and, overall, the treatment was considered to be very safe [74,77,79,81]. In their systematic review on psilocybin, Andersen et al. summarized the observations of therapeutic use of psilocybin in a total of 145 patients and described the following adverse drug reactions: Transient anxiety or fear (27%), headache (22%), nausea or purging (12%), psychological discomfort (11%), experiences of autobiographic hallucination (10%), physical discomfort (8%), transient paranoid ideation (3%), transient thought disorder (1%), insomnia (1%), diarrhea (1%), and strong non-responsive dissociative state (*n* = 1, 1%). No cases of flashbacks or HPPD were observed [74].

Similar to treatment efficacy, it will be of significant interest to compare side effects of psilocybin treatment to those of established treatment options. In the above-mentioned trial comparing psilocybin vs. escitalopram, the percentage of adverse events was nearly identical, with 87% in the psilocybin group and 83% in the escitalopram group. Serious adverse events, psychotic symptoms, or visual perceptual changes occurred in neither of the two groups. The most common adverse events were also comparable: headaches occurred in 67% of the participants in the psilocybin group and in 52% in the escitalopram group, and nausea occurred in 27% of the psilocybin group and 31% of the escitalopram group [81].

In a double-blind placebo-controlled study on the antidepressant and anxiolytic effects of psilocybin in cancer patients (*n* = 51), common physical effects after the administration of psilocybin were transient, dose-dependent increases in blood pressure, and, in some cases, nausea and headaches [77].

**Figure 2:**
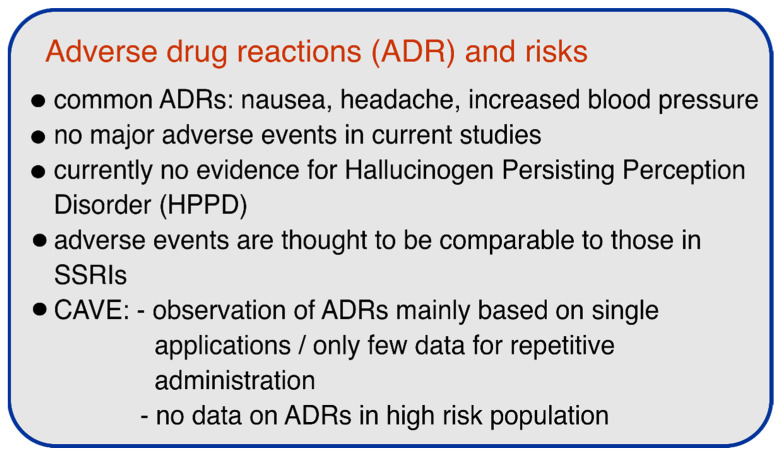
Adverse drug reactions (ADRs) and risks of psilocybin.

According to the available controlled trials, the use of psilocybin appears to be relatively safe and no serious adverse events or cases of HPPD were reported. However, it is notable that most of the reported and ongoing studies excluded patients with schizophrenic disease, major depressive disorder with psychotic features, or severe personality disorder, due to the recommended guidelines to minimize potential risks in psychedelic research [94]. Although this exclusion of at-risk patients in trials is a necessary first step and safety measure, further studies must address the safety of psilocybin or other psychedelics in patients to facilitate the implementation of these substances in clinical practice and to ensure a safe therapeutic environment. Figure 2 summarizes the most important safety aspects.

### 3.4. Limitations of Current Research, Challenges and an Outlook

Similar to other studies and clinical trials investigating new pharmacological treatment options, it must be considered that the participants are highly selected, and patients with a high risk of suicide, a history of psychosis, a positive family history of schizophrenia, or severe depression with psychotic features or severe personality disorders were excluded in the current studies on psilocybin. Although it is reasonable to avoid administrating a 5-HT_2A_-agonist to a person suffering from a schizophrenic disorder, there is currently no evidence on which patient population may benefit the most from psilocybin therapy, and for which patient population it may be strictly contraindicated. Thus, it is will remain a subject of future research to assess the treatment response under psilocybin in different subtypes of MDD, including long-term recurrence rates after a successful treatment and remission.

Another major concern in studies containing a substance with some kind of “fame” such as psilocybin or MDMA, is their susceptibility to selection bias, because patients participating in trials often look forward to the treatment with the respective substance. Some participants may even have prior experience in the use of psychedelics, amplifying a positive expectancy. This has to be considered particularly in studies using self-rated measurements. Assessing the efficacy of psilocybin in placebo-controlled studies is also challenging due to its strong subjective mind-altering effects. There have been attempts to introduce an active placebo, e.g., with niacin, that often causes flushing similar to psilocybin. Nevertheless, due to the lack of psychedelic experience under niacin, it is highly likely for the participants to determine whether they received the verum or the placebo. The insufficient drug blinding is also relevant for the study staff and psychotherapists accompanying the study session [29,76], because both patients and therapists are subject to potential expectation bias [95]. Thus, some studies used a cross-over design that, however, is less able to reliably differentiate drug effects from spontaneous remission during long-term monitoring. An alternative approach that was used in several studies was to administer a dose of psilocybin that was supposed to be sufficiently small to be subtherapeutic. However, there is still no reliable evidence for subtherapeutic versus therapeutic doses, and drug effects of psilocybin within so-called microdosing have been previously described [37]. Accordingly, antidepressant effects and improved fear extinction learning have been observed after administration of Dimethyltryptamine (DMT) at low doses in an animal model [96].

The beneficial setting and therapeutic guidance during the sessions may not only improve safety and therapeutic effects, it may also attenuate the possibility of differentiating between beneficial effects of the substance and beneficial effects of the therapeutic alliance, and enable clarification of the synergistic effect between drug and therapist. It is well known that a good therapist–patient relationship and strong therapeutic alliance are by themselves strong therapeutic factors that contribute to antidepressant effects [97].

In addition, the necessary special setting and, at least in the actual studies, the intense therapeutic support by two specially trained therapists (usually a male/female dyad for up to 6 h) may also raise economic questions. Finally, considering the above-mentioned importance of “set” and “setting” for the psychedelic experience, and the associated long-term outcome and therapeutic benefit, suggests the development of screening tools to identify patients who may benefit from a psilocybin therapy and those who may not. For a thorough review on the biases and difficulties in conducting studies with psychedelic drugs, see Sellers et al. [98].

Several studies are ongoing to overcome some of the limitations mentioned and to shed light on some of the outstanding issues. One double blind phase 2 trial conducted by the Usona Institute on psilocybin in the treatment of major depressive disorder is investigating 80 participants under the two conditions of a verum group receiving psilocybin 25 mg and an active placebo control with 100 mg niacin [99]. The COMPASS Pathways study aims to gain information on the optimal dose of psilocybin in treatment-resistant depression. This study will compare the effects of 25 mg vs. 10 mg vs. 1 mg of psilocybin [100], and also aims to shed further light on the role and efficacy of microdosing. Another multicenter phase II trial called EPIsoDE will include 144 patients with treatment-resistant depression in a cross-over design [101].

Nevertheless, although these studies involve a relatively large number of participants and aim to provide more information on safety and dosing of psilocybin treatments, several caveats must be addressed in future investigations.

## 4. Conclusions

Numerous studies suggest beneficial effects of psilocybin in the treatment of depression. These effects appear to be long lasting, with overall good safety and tolerability provided psilocybin is administered in a safe and professional environment. However, the detailed mechanism of action of psilocybin is still not fully understood. Recent hypotheses suggest a promotion of neural plasticity and alterations in neural network functioning, presumably due to the 5-HT_2A_ agonism of psilocybin. In addition, internal factors such as the expectation of the person receiving the substance, and external factors such as the environment in which psilocybin is administered, are thought to be crucial for its efficacy and safety. However, the importance of the “psychedelic” experience remains controversial and is still subject to further research. The insights gained in recent research on psilocybin are considerable and provide hope for new treatment strategies for mood disorders. Nevertheless, this perspective requires further research, with careful consideration of the precautions, to reliably achieve a potential therapeutic effect while minimizing the risk for potential harm.

## Data Availability

Data sharing is not applicable.
